# Influence of pH on the Kinetics and Products of Photocatalytic Degradation of Sulfonamides in Aqueous Solutions

**DOI:** 10.3390/toxics10110655

**Published:** 2022-10-29

**Authors:** Dominika Sapińska, Ewa Adamek, Ewa Masternak, Wioleta Zielińska-Danch, Wojciech Baran

**Affiliations:** Department of General and Analytical Chemistry, Faculty of Pharmaceutical Sciences in Sosnowiec, Medical University of Silesia in Katowice, Jagiellońska 4, 41-200 Sosnowiec, Poland

**Keywords:** sulfonamides, ecotoxicity, photocatalysis, degradation products, mechanism

## Abstract

The aims of the study were to determine the kinetics of the photocatalytic degradation of six sulfonamides in the presence of TiO_2_-P25 in acidic, neutral, and alkaline solutions and to identify the structures of the stable products. It was stated that the pH of the solution significantly affected the photocatalytic degradation rate of sulfonamides in acidic and alkaline environments, and the effect likely depended on the susceptibility of sulfonamides to attack by hydroxyl radicals. In the post-reaction mixture, we identified the compounds resulting from the substitution of the aromatic rings with a hydroxyl group; the amide hydrolysis products; the hydroxylamine-, azo, and nitro derivatives; and the compounds formed via the elimination of the sulfone group. Moreover, previously unknown azo compounds were detected. Some degradation products of sulfonamides may exhibit marked bacteriostatic activity and high phytotoxicity. The azo and nitro compounds formed in an acidic environment may be potentially more toxic to aquatic ecosystems than the initial compounds.

## 1. Introduction

Sulfonamides (SNs), sulfanilamide derivatives (SADs), are antimicrobial drugs widely used particularly in veterinary medicine. More than 70% of global antimicrobial use has been estimated to be in livestock. In 2020, the antibiotics sales for food-producing animals in 31 European countries and the United States reached more than 5500 t and 10,500 t, of which SNs were 9.9% and 7.2%, respectively [1,2]. After use, both the unmetabolized SNs as well as their metabolites are released directly into the environment. These drugs are quite persistent and nonbiodegradable. Therefore, a significant proportion of them remains biologically active in the environment over a long time [3]. For example, almost 50 mg of SNs per kg of dry weight was detected in the manure from livestock farms [4]. In recent years, only a slight decrease in SN consumption has been observed [1,2]. SAD derivatives are also introduced into the environment as herbicides. Although in 2011 the European Commission decided not to re-register Asulam (methyl (4-aminobenzene-1-sulfonyl) carbamate), it is still manufactured and used in many countries [5,6,7,8,9].

SNs exhibit bacteriostatic effects, but their toxic concentrations to microorganisms are relatively high. According to Ferrari et al. [10], the determined EC_50_ values of sulfamethoxazole (SMX) against the *Vibrio fischeri* strain ranged from 43 to 53 mg/L. In turn, the IC_50_ value of SMX against *Arthrobacter globiformis* was >127 mg/L [11]. The sensitivity of invertebrates and vertebrates to SMX is also not high [10,12,13,14]. On the other hand, the EC(IC)_50_ values of SMX against algae (*Scenedesmus vacuolatus*) were about 1.5 mg/L, and for higher plants (*Daucus carota*), even <0.05 mg/L [15,16].

These data indicate that SNs are highly phytotoxic. Thiele-Bruhn and Beck reported that soil biocenoses were even more sensitive to this antibiotics group [17]. Another type of risk is related to the long-term exposure of environmental microorganisms to sub-toxic concentrations of SNs. This may result in the emergence of antibiotic-resistant genes (ARGs) and their potential transfer to pathogens [18]. For this reason, the treatment of wastewater containing SNs using biological methods is also risky [19].

An attempt to solve the problem described above is to use a photocatalytic process to remove SNs from wastewater [20,21,22,23]. Hydroxyl (HO^•^) and superoxide (O_2_^−•^) radicals are generated during the irradiation of aqueous solutions containing a photocatalyst. The presence of free radicals can be confirmed by analyzing the products of their reaction, e.g., HO^•^ with benzene, or using the ESR method [24,25,26,27]. Currently, experiments on extending the useful irradiation spectrum in the heterogenous photocatalysis are carried out [28,29]. They even initiate the decomposition of non-biodegradable organic compounds [30,31,32,33,34,35,36,37,38]. In addition to the effective degradation of antibiotics, the photocatalytic process also affects the inactivation of ARGs, the disinfection of the processed material, and sometimes the complete mineralization of pollutants [34,39]. It was shown that even a short-term irradiation of the solutions in the presence of photocatalyst(s) causes a significant decrease in the concentration of the degraded antibiotic, although its transformation products remain in the post-reaction mixture. Simultaneously, an inhibition of antimicrobial activity in antibiotic solutions was observed [40]. However, the low susceptibility of organisms used in popular toxicity tests to SNs can be a serious problem in the assessment of ecotoxicological effects. As a consequence, in order to observe the toxic effects of the initial solutions of SNs, their concentrations used in the tests must be much higher than those observed in the environment [4]. In addition, the concentrations of the decomposition products of SNs must be high enough to be able to detect their potential toxicity.

Wastewater containing antibiotic residues, including SNs, may be slightly alkaline (livestock wastewater) but may also be close to neutral (municipal wastewater) or acidic (industrial wastewater). This fact should be taken into account when planning the use of photocatalysis for wastewater treatment. The pH has been shown to affect the dynamics of photocatalytic degradation of SNs [35,41,42]. Therefore, pH values likely play a significant role in the degradation pathways of these antibiotics. The effect of pH on free radicals generation during the photocatalytic process in the presence of TiO_2_ was thoroughly described [38,43,44].

The aims of the study were to determine the kinetics of the photocatalytic degradation of six SNs (Table 1) in the presence of TiO_2_-P25 in acidic, neutral, and alkaline aqueous solutions and to identify the structures of the stable products. Based on these experiments, the effect of pH on the mechanism of SN degradation and the potential toxicity of the products were assessed.

## 2. Materials and Methods

### 2.1. Reagents

The characteristics of the SNs used in the experiments are presented in Table 1.

In addition, TiO_2_-P25 (Evonik Aeroxide^®^), HCl and NaOH solution (p.a.; 1 mol/L; POCH), water (LC-MS CHROMASOLV^®^; Fluka-Analytical, Buchs, Switzerland), acetonitrile (LC-MS CHROMASOLV^®^; Fluka-Analytical, Buchs, Switzerland), and formic acid (p.a. SIGMA-ALDRICH, St. Louis, MO, USA) were used in the experiments.

### 2.2. Photocatalytic Process

The stock solutions (0.1 mmol/L) of six SNs (Table 1) were prepared in redistilled water. A 150 mL solution of each SN was placed in a glass beaker with a glass barbotage tube, and 75 mg of TiO_2_-P25 was added. In the dark, the pH of each sample was adjusted to the expected value with HCl or NaOH solutions. The mixtures in the beakers were irradiated from above with fluorescent lamps (TL-K 40W Actinic BL; Philips). The intensity of irradiation absorbed by the mixtures was 0.37 W/L. Throughout the experiment, the samples were stirred with compressed air.

### 2.3. Samples Analysis

Aliquots for analysis were removed from the mixtures prior to irradiation and after a specific time. They were filtered (CHROMAFIL RC-20/25; Macherey Nagel) directly into glass vials and immediately analyzed using an Acquity UPLC/DAD system coupled with Xevo G2-XS-QTOF (Waters). The degradation products of SNs were separated using an Acquity UPLC BEH C18 column, 100 × 2.1 mm (Waters), and the mobile gradient phase consisted of a mixture of acetonitrile and water at 35 °C. The DAD detector recorded peaks at 272 nm, while the QTOF detector operated sequentially in ES+ MS and ES+ MS/MS modes. The details of the analysis procedure are presented in Table 2.

The injection volumes of the sample were 1 µL and 5 µL during the quantitative and qualitative analyses, respectively.

### 2.4. Analysis of the Results

The kinetics of SN degradation were assessed based on the peaks recorded using the DAD detector. For each experiment, a linear regression model of the relationship ln C_0_/C = f(t) was determined, where the C_0_/C is the peak area quotient of SNs in the initial solution and in the solution after irradiation time t. This procedure is correct and common for pseudo-first-order reactions.

Molecular formulas of the degradation products of the SNs were determined based on the monoisotopic masses of molecular ions (M+H^+^) obtained using the MS/TOF technique with ES+ ionization. Structural formulas of the SN degradation products were proposed based on their molecular formulas, and fragmentation spectra were determined using the MS/MS/QTOF technique with ES+ ionization, at collision energies from 10 to 25 V. Aliphatic degradation products of the SNs were not identified.

One of the products of SN transformation is SAD, which is hydrolyzed to sulfanilic acid during ES+ ionization. Therefore, this compound was additionally identified based on the retention time (t_R_).

### 2.5. Toxicity Prediction

The potential ecotoxicity of the identified degradation products was estimated only for SMX using ECOSAR (Ecological Structure Activity Relationships) Application 2.2. The ecotoxicity prediction also extended to the aliphatic degradation products of SMX, which were not identified during the chromatographic analysis but are described in the literature.

## 3. Results

### 3.1. Kinetics of SN Degradation

**Figure 1:**
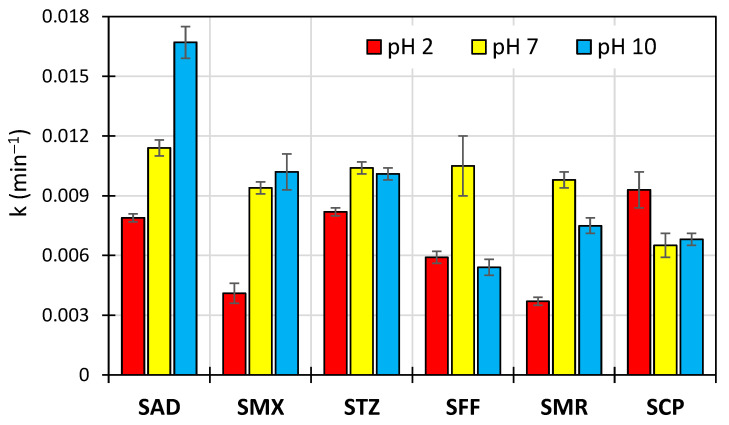
Reaction rate constants of the photocatalytic degradation of SNs in acidic, neutral, and alkaline environments.

In all the performed experiments, the determined relationship lnC_0_/C = f(t) was the linear function with a coefficient of determination (R^2^) > 0.9. This indicated that the photocatalytic degradation of SNs could be described using pseudo-first order kinetics. The experimentally found values of the reaction rate constant (k) are shown in Figure 1.

Under the constant experimental conditions, there was no unambiguous and constant relationship between the pH and the degradation rate of SNs. In a neutral environment, the degradation rates of all of the studied SNs were similar, with the exception of SCP. In contrast, in acidic and alkaline environments, the effect varied and depended on the type of SN. Similar observations were already described in previously published articles [41,42].

The photocatalytic degradation of SNs performed in the aqueous environment and in the presence of TiO_2_ can be initiated by photo holes (h^+^), HO^•0^, and depending on the pH, O_2_^−•^ or hydroperoxide radicals (HO_2_^•^) [33,34,35,36,37,38,41]. The participation of h^+^ in a direct reaction with SNs requires the prior adsorption of the reactants onto the photocatalyst surface. The lifetime of HO^•^ radicals generated on the TiO_2_ surface is extremely short (~10^−9^ s). This indicates that in the case of these radicals, a prior adsorption of the reactants is necessary [41]. Therefore, the photocatalytic reaction rate in the presence of TiO_2_ suspension should mainly depend on the degree of adsorption of the reactants.

SNs have amphoteric properties (Table 1). However, at a pH of 10–11, the TiO_2_ surface is negatively charged, and the SNs exist as organic anions. Under these conditions, an electrostatic repulsion between catalyst particles and reagent molecules occurs, and the photocatalytic reaction should be inhibited. A similar phenomenon should be observed in solutions at pH 2–3, where the TiO_2_ surface is positively charged and SN molecules exist as cations or neutral molecules; however, we observed no such effect.

In our opinion, the degradation rate of SNs in acidic and alkaline environments is primarily determined by the susceptibility of SN molecules to electrophilic attack by HO^•^ radicals. The stable products of the direct reaction of SNs with HO^•^ identified in acidic solutions (Section 3.2) confirmed our assumptions. Moreover, the lower degradation rate of SNs (except for SCP) in the acidic environment may be associated with the protonation of lone electron pairs in amide and ammonium nitrogen. On the other hand, in an acidic environment, SNs are more susceptible to nucleophilic attack by HO_2_^•^ radicals. They are much less reactive than HO^•^ and have a relatively long half-life but may be responsible for the formation of other products.

**Figure 2:**
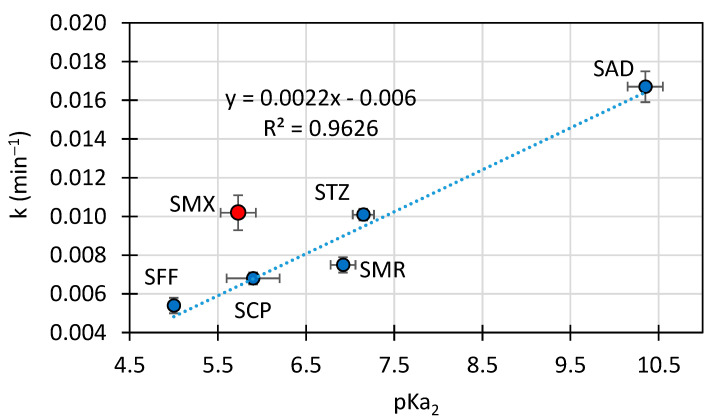
Relationship between pK_a2_ and the degradation rate constant of SNs at pH 10.

Under alkaline conditions, SNs are almost completely dissociated (>99%), with the exception of SAD [46]. A positive linear correlation between the pK_a2_ values and the degradation rate constant (k) in the presence of TiO_2_ P25 was determined for five of the six SNs studied (Figure 2). The SMX result was omitted from the regression equation.

The increase in the pK_a2_ value was a consequence of a lesser electron affinity of the amide group substituent of the studied SNs. Compounds having a lower electron affinity could show higher electron-donor properties. Therefore, this may be the factor that determines the reaction rate of SNs with HO^•^ radicals in the alkaline environment and affects the photocatalytic reaction rates. However, in this case, the photodegradation rate with SMX was higher than expected (Figure 2). The probable cause of this phenomenon is the steric effect. SNs differ from each other not only in the heterocyclic ring but also in the arrangement of the spatial structure [42]. It can affect the degradation rate of these compounds in heterogeneous catalysis. Moreover, SMX differs from other SNs in the ability to form hydrogen bonds in the aquatic environment [47].

### 3.2. Degradation Products

Organic cyclic and aliphatic products, and finally, inorganic compounds were present in the post-reaction solution after the photocatalytic degradation of SNs in the presence of TiO_2_ P25. The simplest compound of the series of the studied SNs was SAD. Tačić et al. [48] proposed its degradation pathway, and aniline, benzoquinone, sulfanilic acid, and aliphatic compounds such as maleic acid, fumaric acid, oxalic acid, acetic acid, formic acid, and inorganic compounds were identified among the products. In turn, according to Dong et al. [49], compounds formed as a result of the attack of HO^•^ radicals on the nitrogen atom of the amide group were present among the degradation products of SAD.

**Figure 3:**
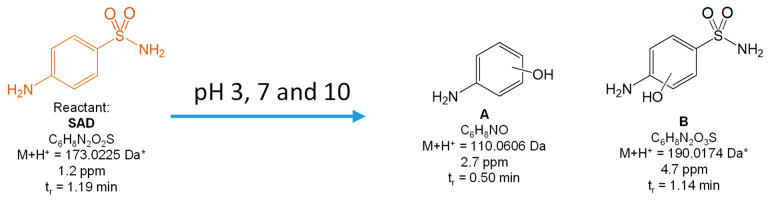
Products of the photocatalytic degradation of SAD after 120 min of UV irradiation in the presence of TiO_2_-P25. (*) primary amide hydrolysis occurs during QTof analysis.

Figure 3 presents the two proposed identified products of SAD degradation, their molecular and structural formulas, the masses of the monoisotopic molecular ions (M+H^+^), and the error in mass determination (in ppm).

Both products were formed as a result of the SAD transformation, regardless of the pH, as a result of the attack by HO^•^ radicals on the aromatic ring of SAD. They were identified immediately after sampling. These compounds may undergo further changes, likely in the presence of oxygen dissolved in water. Because the -OH group is attached directly to the aromatic ring, the products of SAD degradation cannot be a competitive inhibitor of dihydrofolate synthesis; therefore, they should not exhibit antimicrobial activity and phytotoxicity.

**Figure 4:**
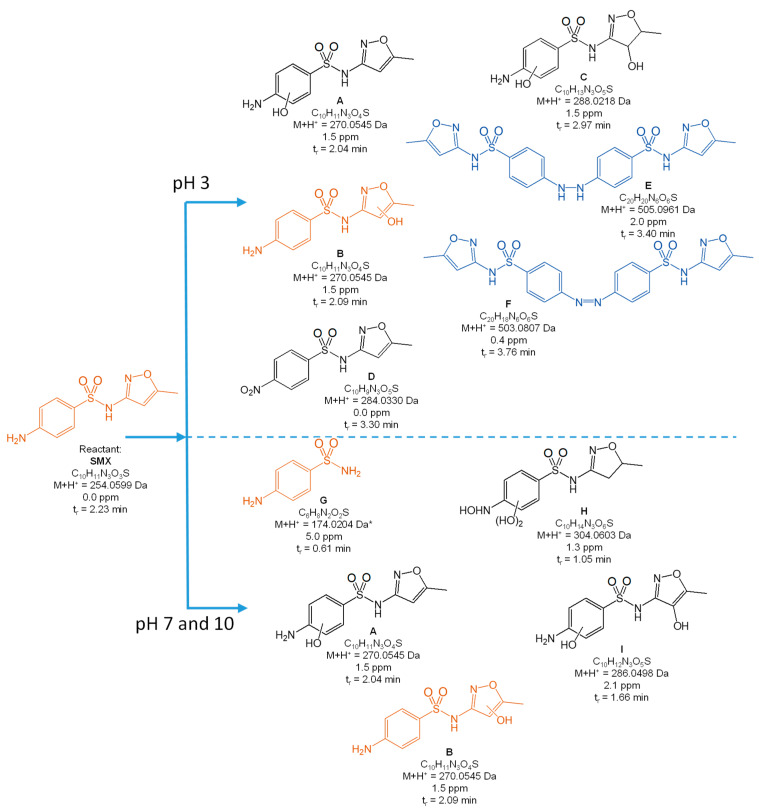
Products of the photocatalytic degradation of SMX after 120 min of UV irradiation in the presence of TiO_2_-P25. (*) primary amide hydrolysis occurs during QTof analysis.

**Figure 5:**
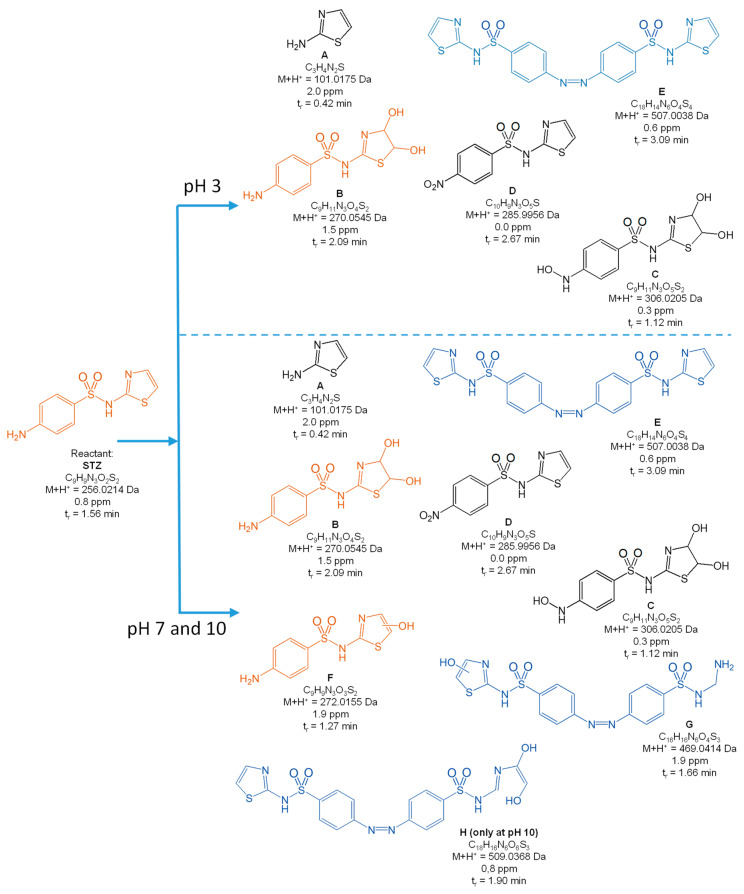
Products of the photocatalytic degradation of STZ after 120 min of UV irradiation in the presence of TiO_2_-P25. (*) primary amide hydrolysis occurs during QTof analysis.

**Figure 6:**
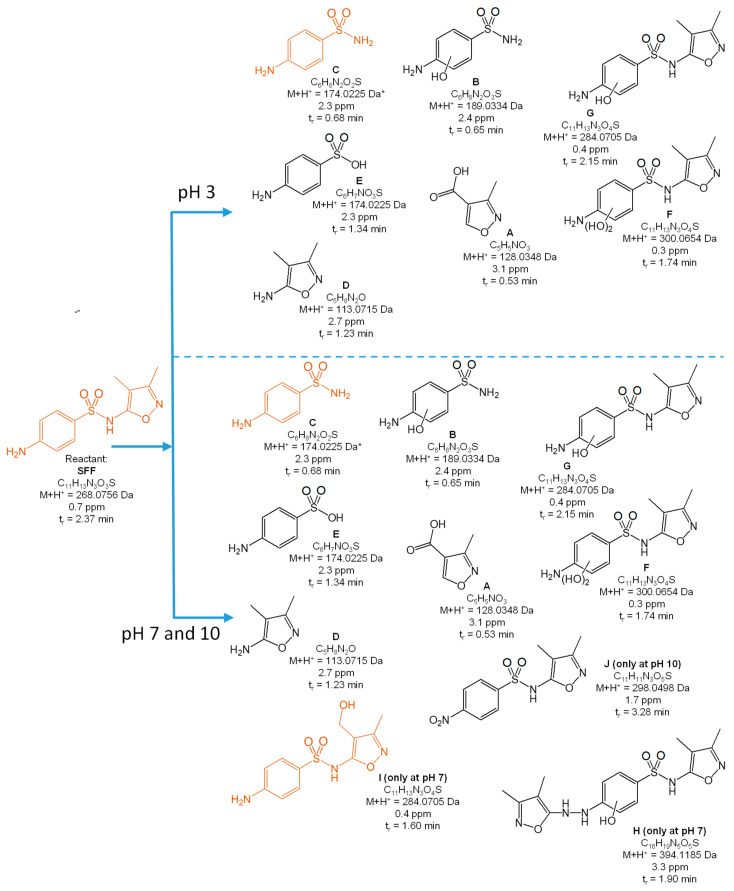
Products of the photocatalytic degradation of SFF after 120 min of UV irradiation in the presence of TiO_2_-P25. (*) primary amide hydrolysis occurs during QTof analysis.

**Figure 7:**
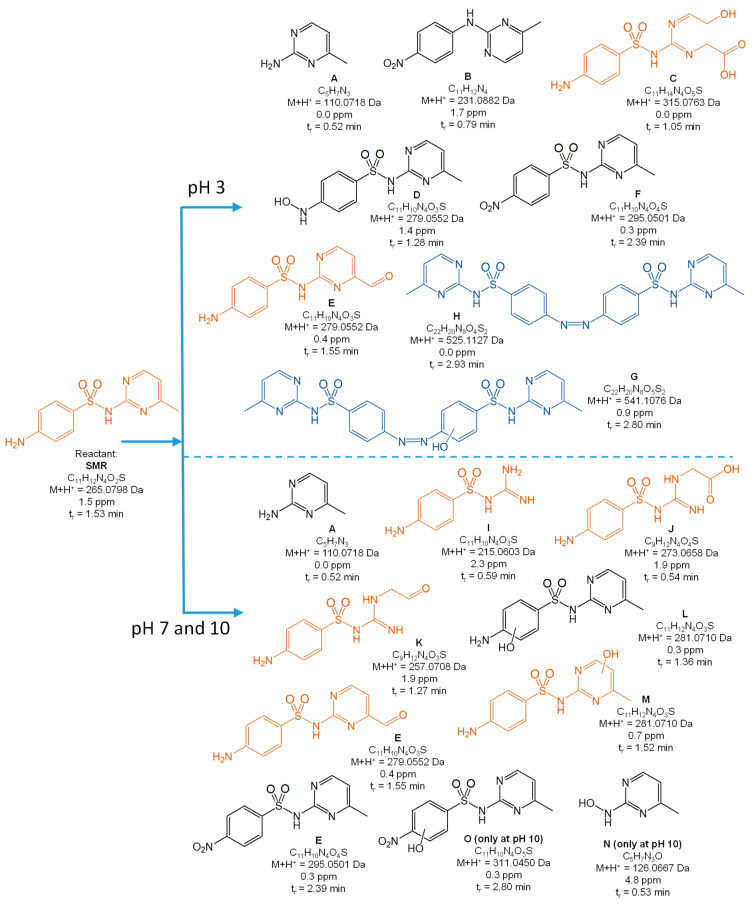
Products of the photocatalytic degradation of SMR after 120 min of UV irradiation in the presence of TiO_2_-P25. (*) primary amide hydrolysis occurs during QTof analysis.

**Figure 8:**
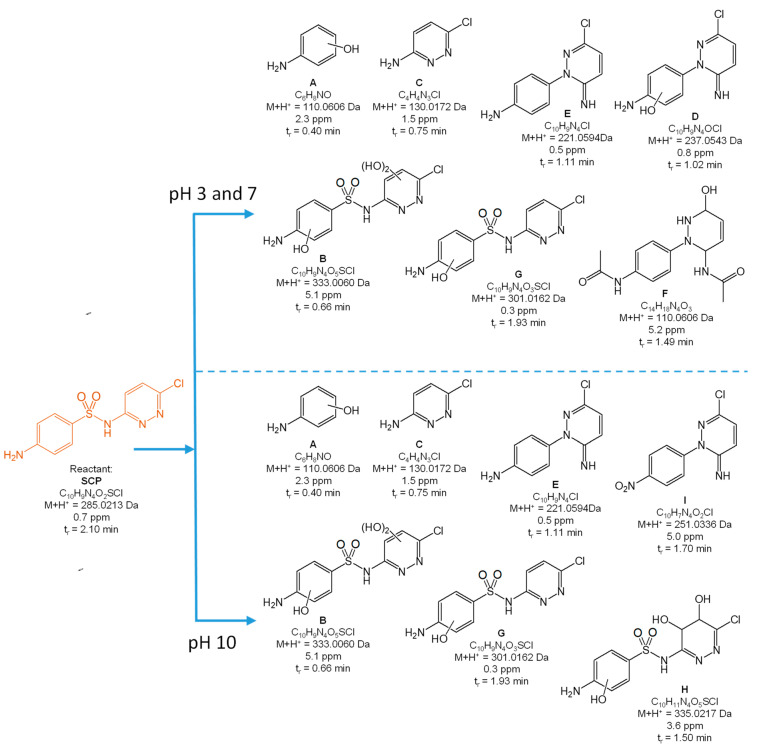
Products of the photocatalytic degradation of SCP after 120 min of UV irradiation in the presence of TiO_2_-P25.

Figure 4, Figure 5, Figure 6, Figure 7 and Figure 8 present the proposed structural and molecular formulas of the degradation products of other SNs after 120 min of irradiation in the presence of TiO_2_-P25. Compounds that may act as competitive inhibitors of dihydrofolate synthesis are marked in orange. Compounds that can be converted to the active form in the environment are marked in blue.

The analysis of the results (Figure 4, Figure 5, Figure 6 and Figure 7) indicated that the qualitative composition of the solutions after SN degradation did not differ, with few exceptions, in the neutral and alkaline environments. More differences were found in the acidic environment. Under these conditions, the compounds with the hydroxyl group in the aromatic and/or heterocyclic ring and those resulting from amide hydrolysis were identified as the main products. Similar compounds were also reported by other researchers [41,50,51,52,53]. These compounds were formed as a result of the attack by HO^•^ radicals in regions with higher electron density in SN molecules [54].

The compounds formed as a result of the oxidation of the amino group to -NHOH and -NO_2_ and those containing a degraded and oxidized heterocyclic ring were found among the products of photocatalytic degradation. More interesting is the fact that previously unknown azo compounds were also detected. These products were found in the solutions of SMX (Figure 4E,F), STZ (Figure 5E,G,H), and SMR (Figure 7G,H) and were present at pH 3. In the case of STZ, they were also detected in the alkaline environment. The azo products could derive from the recombination of organic radicals (cation radicals) generated in solutions. The generation of cation radicals is more likely in an acidic environment. Bhat and Gogate reported the mechanism of their formation as a result of the reactions initiated by O_2_^−•^ radicals [54]. The azo derivatives may undergo transformation in the environment to the initial SNs.

Other products were identified in solutions after the photocatalytic degradation of SCP (Figure 8). Compounds D, E, F, and I resulted from the elimination of the sulfone group. In turn, a similar product was identified in the post-reaction SMR solution (Figure 7B). Shah and Hao presented the compounds formed during sulfamethoxypyridazine degradation as having similar structures [55]. They proposed a mechanism and the thermodynamic aspects of the HO^•^-initiated reactions. In our opinion, an important stage in this process is the generation of the (NH_2_)C_6_H_6_^•^ radical as a result of an attack by the O_2_^−•^ radical and the breakage of the C-S bonds. The stable hydrogen sulfate(VI) anion can also form in this reaction.

As mentioned above, the majority of the degradation products formed in reactions of SNs with HO^•^ radicals. This was consistent with the assumption that the susceptibility of SNs to reactions with these radicals may be of key importance when considering the degradation rate.

### 3.3. Toxicity Prediction of the Degradation Products of SMX

**Figure 9:**
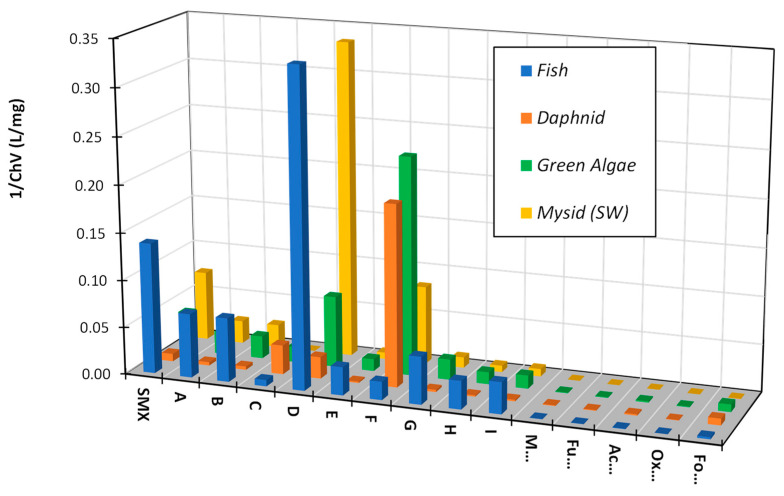
Predicted chronic toxicity of SMX, of its photocatalytic degradation products (compounds marked with letters A through I in Figure 4), and of maleic (M…), fumaric (Fu…), acetic (Ac…), oxylatic (Ox…), and formic (Fo…) acids to selected aquatic organisms using the ECOSAR model.

Most of the products described in Section 3.2 (Figure 4, Figure 5, Figure 6, Figure 7 and Figure 8, marked in orange) contain a moiety that is responsible for the biological activity of SNs. However, it is not excluded that other photocatalytic degradation products of SNs are also hazardous to the environment. Figure 9 shows the predicted chronic toxicity of the products identified in solutions after the photocatalytic degradation of SMX and the aliphatic degradation products of SNs reported in the literature [48]. Chronic aquatic toxicity (ChV) inversion was used as a measure of toxicity to representative environmental (aquatic) organisms (unicellular and multicellular). ChV is defined as the geometric mean of the no-observed-effect concentration (NOEC) and the lowest-observed-effect concentration (LOEC).

**Figure 10:**
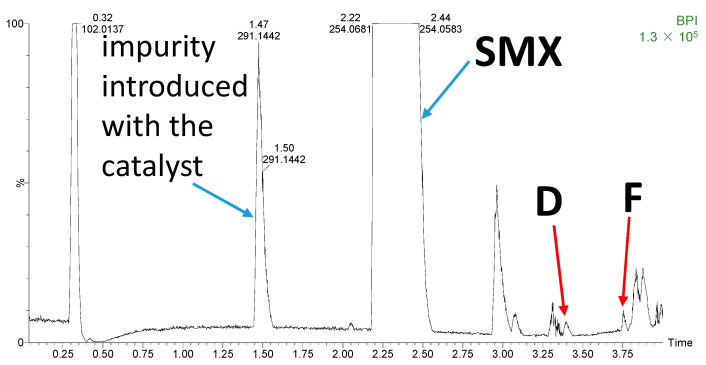
Chromatogram of SMX solution after 120 min of irradiation in the presence of TiO_2_ P25 at pH 3.

The predicted results of the ECOSAR model indicated that substances formed during the photocatalytic degradation of SNs may be highly toxic to aquatic organisms, e.g., the products containing the nitro group (D) and the azo bond (F). However, the estimated content of toxic substances in the analyzed samples was very low (Figure 10).

The complete mineralization of antibiotics via photocatalysis is much slower than their degradation and the inactivation of antimicrobial activity, because it requires a significantly longer irradiation time and is ineffective in real wastewater [40]. For these reasons, the irradiation time necessary to achieve the inhibition of antimicrobial activity in solutions containing antibiotics residues was sufficient. We showed that the aliphatic compounds remaining in solution were likely non-toxic or little toxic to environmental aquatic organisms (Figure 9). In addition, subtoxic amounts of SNs to bacteria and their biologically active derivatives also remained in the solutions after irradiation (Figure 4, Figure 5, Figure 6, Figure 7 and Figure 8). The high phytotoxicity of these compounds to aquatic plants could be a serious threat, especially to vulnerable ecosystems. A complete assessment of the photocatalytic efficiency of SN degradation should include ecotoxicity tests with the use of plant indicators; however, the long testing time is a major disadvantage.

## 4. Conclusions

The pH of the solution significantly affected the photocatalytic degradation rate of SNs in the presence of TiO_2_ P25. This effect was particularly evident in the acidic and alkaline environments and likely depended on the susceptibility of SNs to attack by HO^•^ radicals. The pH did not affect the type of degradation products of SAD. In the case of other SNs, there were significant differences among the products identified in acidic, neutral, and alkaline solutions. In the post-reaction mixture, we identified the compounds resulting from the substitution of the aromatic rings with a hydroxyl group; the amide hydrolysis products; the hydroxylamine, azo, and nitro derivatives; and the compounds formed via the elimination of the sulfone group. The azo and nitro compounds may be potentially more toxic to aquatic ecosystems than the initial SNs. Some degradation products of SNs may exhibit marked bacteriostatic activity and high phytotoxicity. The biological activity analysis of the products is an important determinant to assess the effectiveness of the treatment of wastewater containing SN residues using the photocatalytic method. In turn, the pH change of the photocatalytic process to increase the treatment efficiency is not justified.

## Figures and Tables

**Table 1:** Characteristics of the studied SNs.

Name/CAS	Abbreviation	Structural Formula	Purity/Manufacturer	pKa_1_^a^	pKa_2_^a^
Sulfanilamide 63-74-1	SAD	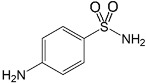	98.0%; Fluka	6.92 ± 0.14	10.35 ± 0.21
Sulfamethoxazole 723-46-6	SMX	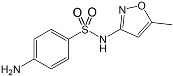	99.0%; Fluka	1.86 ± 0.32	5.73 ± 0.20
Sulfathiazole 72-14-0	STZ	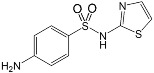	99.0%; Sigma-Aldrich	2.22 ± 0.27	7.15 ± 0.12
Sulfisoxazole 127-69-5	SFF	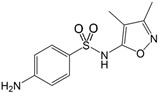	99.0%; Sigma-Aldrich	1.69 ± 0.27	5.0 ± 0.0
Sulfamerazine 127-79-7	SMR	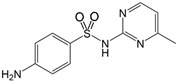	99.0%; Sigma-Aldrich	2.21 ± 0.15	6.92 ± 0.14
Sulfachloro-pyridazine 80-32-0	SCP	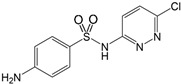	98.0%; Sigma-Aldrich	2 ± 3 ^b^	5.90 ± 0.30 ^b^

^(a)^ [42]; ^(b)^ [45].

**Table 2:** Chromatographic separation conditions.

	Eluent Gradient		
Time (min)	H_2_O with 0.01% HCOOH	CH_3_CN with 0.01% HCOOH	Flow Rate (mL/min)
For SAD			
0	99.9%	0.1%	0.30
2	99.9%	0.1%	0.30
2.5	99.0%	1.0%	0.30
3	80.0%	20%	0.40
3.5	80.0%	20%	0.40
4	99.9%	0.1%	0.30
For other SNs			
0	95%	5%	0.35
3.0	60%	40%	
3.3	20%	80%	
3.5	95%	5%	

## Data Availability

The data presented in this study are available upon request from the corresponding author. The data are not publicity available due to the very large sizes of the chromatographic files.
